# IL-33 and ST2 levels in chronic kidney disease: Associations with inflammation, vascular abnormalities, cardiovascular events, and survival

**DOI:** 10.1371/journal.pone.0178939

**Published:** 2017-06-14

**Authors:** Ozkan Gungor, Hilmi Umut Unal, Aydin Guclu, Mustafa Gezer, Tayfun Eyileten, Fatma Betül Guzel, Orcun Altunoren, Ertugrul Erken, Yusuf Oguz, Ismail Kocyigit, Mahmut Ilker Yilmaz

**Affiliations:** 1 Department of Nephrology, Sütçü İmam University, Kahramanmaras, Turkey; 2 Department of Nephrology, Gulhane School of Medicine, Ankara, Turkey; 3 Department of Nephrology, Ahi Evran University, Kirsehir, Turkey; 4 Department of Nephrology, Erciyes University, Kayseri, Turkey; The University of Tokyo, JAPAN

## Abstract

**Objective:**

Increased inflammation, associated with the increase in chronic kidney disease (CKD) stage, has a very important influence in vascular injury and cardiovascular diseases. In this study, we aimed to investigate the levels of IL-33 and ST2 in the different stages of CKD and to determine their effect on vascular damage and cardiovascular events (CVE).

**Methods:**

This was an observational cohort study in which serum IL-33 and ST2 were obtained from 238 CKD (stages 1–5) patients. We examined the changes in IL-33/ST2 levels in CKD patients, as well as the association with a surrogate of endothelial dysfunction. Fatal and non-fatal CVE were recorded for a mean of 24 months. We also performed a COX regression analysis to determine the association of IL-33/ST2 levels with CVE and survival.

**Results:**

IL-33 and ST2 levels were significantly increased and estimated glomerular filtration rates (eGFR) were decreased. Flow-mediated dilatation (FMD) was significantly decreased from stage 1 to stage 5 CKD. IL-33 and ST2 levels were associated with FMD, and ST2 was a predictor. Multivariate Cox analysis showed that the presence of diabetes mellitus, smoking, and proteinuria and haemoglobin, Hs-CRP, IL-33, and ST2 were associated with the risk of CVE. Kaplan-Meier survival curves showed that patients with IL-33 and ST2 levels below the median value (IL-33 = 132.6 ng/L, ST2 = 382.9 pg/mL) had a higher cumulative survival compared with patients who had IL-33 and ST2 levels above the median value (log-rank test, p = 0.000).

**Conclusion:**

This is the first study that demonstrates that serum IL-33 and ST2 are associated with vascular injury, cardiovascular events, and survival in CKD patients.

## Introduction

The prevalence of chronic kidney disease (CKD) in epidemic proportions of the population has increased at a rate of 10.6% to 13.4% [1]. Chronic renal failure is a progressive disease. While the transition between stages can sometimes be very fast, it may, in other cases, take years. The most important factors influencing this course are hyperglycaemia, hypertension, high protein diet, and inflammation [2]. Inflammation is an important factor that may play a role in the progression of chronic renal failure and in the progression of other possible complications associated with chronic renal failure [3]. In past studies, it has been shown that inflammation increases in parallel with increases in CKD stage. While the renal functions decline, the circulating biomarker levels of systemic inflammation—like the CRP, pentraxin-3 (PTX3), interleukin-6 (IL-6), and the anti-inflammatory IL-10—increase constantly [4–6].

Inflammation has an extremely important influence in cardiovascular diseases. A number of studies have been conducted on this, and they report the significance of the pro-inflammatory mediators in the development and progression of several cardiac diseases like heart failure [7,8]. Vascular damage and cardiovascular events are the most important causes of morbidity and mortality in chronic renal failure [9]. Additionally, advanced age, oxidative stress, smoking, hyperlipidaemia, and hypertension are among other factors that increase this damage [10]. According to recent research, persistent inflammation is among the major reasons for general premature and vascular aging. It has been assumed that allostatic overload may contribute to increased oxidative stress levels as well as the activation of the innate immune system, sympathetic-vagal imbalance and low-grade persistent inflammation [11]. C-reactive protein (CRP) and pro- and anti-inflammatory cytokines, which are among the well-known inflammatory markers, have a correlation with the underlying reasons and results of inflamed uremic phenotypes (like oxidative stress, endothelial dysfunction, vascular calcification, and cardiovascular disease). [12,13].

As a new member of the IL-1 superfamily, Interleukin (IL)-33, behaves like a nuclear factor, cytokine, and as a dual function molecule. IL-33 is known as the ST2 receptor’s (ST2L) functional ligand. It binds ST2L to the inflammatory cell membranes. This binding leads to activation of mitogen-activated protein kinase (MAPK)-kinases, and additionally, some other biochemical pathways also become activated [14,15]. This leads to the subsequent activation of inhibitor of nuclear factor-κB (NF-κB) kinase (IKK) complex; and this, in turn, activates NF-kB and causes it to exert its pro-inflammatory activities [16]. In CKD, IL‑33 plays a crucial role and might lead to the progression of renal fibrosis, which is related to renal graft damage and lupus nephritis [17]. Furthermore, IL‑33 also contributes to acute kidney injuries [18]. Several studies have been conducted on animals, and the reports show that IL-33/ST2 has an active role in cardiovascular diseases [19,20]. In addition, soluble ST2 (sST2) has a future value as a biomarker to predict the worse outcomes in some cardiovascular diseases [21].

In this study, for the first time in literature, we aimed to investigate the levels of IL-33/ST2 in the different stages of chronic kidney disease and to determine their effects on vascular damage and cardiovascular outcomes.

## Methods

### Patients and study design

Between July 2013 and January 2016, 408 patients were referred to the renal unit of the Gulhane School of Medicine Medical Center, Ankara, Turkey for the first time because of suspected or manifest CKD. All patients included in the study were diagnosed as having CKD according to the National Kidney Foundation K/DOQI guidelines [22]. Seventy-seven cases were excluded based on the exclusion criteria, including acute infections and unwillingness to participate in the study. Ninety-three eligible patients dropped out for the following reasons: they were lost to follow-up (n = 50) or withdrew consent (n = 43). Stages of CKD were determined using estimated glomerular filtration rates (eGFR), which were calculated via the Modification of Diet in Renal Disease (MDRD) Equation [23]. Two hundred and thirty-eight patients were included in the final analysis. None of the patients in stage 5 CKD were on haemodialysis or peritoneal dialysis.

All included patients were followed-up with for time-to-event analysis until the occurrence of fatal or nonfatal cardiovascular events. Fatal and nonfatal CVE including death, stroke, and myocardial infarction were recorded. A local ethical committee (at the Gulhane School of Medicine) approved the study protocol, and all patients were included in the study after signing informed consent forms.

### Biochemical analyses

All blood samples were obtained from patients in the morning after 12 hours of fasting for the evaluation of fasting plasma glucose (FPG), serum albumin, total serum cholesterol (TC), triglyceride (TG), and high-density lipoprotein (HDL) and low-density lipoprotein (LDL) cholesterol. Total serum cholesterol, TG, and HDL-cholesterol were measured by enzymatic colorimetric method with an Olympus AU 600 automated analyzer using reagents from Olympus Diagnostics, GmbH (Hamburg, Germany). LDL-cholesterol was calculated by Friedewald’s formula [24]. For the measurement of high-sensitive C-reactive protein (Hs-CRP), serum samples were diluted with a ratio of 1/100 with the diluent solution. Calibrators, kit controls, and serum samples were all added to each micro well with an incubation period of 30 minutes. After three washing intervals, 100 μL enzyme conjugate (peroxidase labeled anti-CRP) was added to each micro well for an additional 15 minutes’ incubation at room temperature in the dark. The reaction was stopped with a stop solution and photometric measurement was performed at the 450 nm wavelength. The amount of serum samples was calculated as mg/L with a graphic that was made by noting the absorbance levels of the calibrators. The serum basal insulin value was determined by the coated tube method (DPC-USA). An insulin resistance score (i.e., Homeostasis Model Assessment-Insulin resistance (HOMA-IR)) was computed using the following formula: HOMA-IR = fasting plasma glucose (mg/dL) x Immunoreactive insulin (IRI) (μIU/mL) / 405. Proteinuria was quantified using 24-hour timed urine collection [25].

Serum total calcium was measured by the Cresolphtalein complex one method using Menagent Calcium 60sec kits (Menarini Diagnostics, Florence, Italy). Serum phosphorus was measured by the ammonia molybdate complex method using Menagent Phosphofix kits (Menarini Diagnostics, Florence, Italy). Intact parathormone was measured using IRMA, using a commercial kit (Immulite Intact PTH) from Diagnostic Product Corporation (Los Angeles, CA) with a sensitivity of 1 pg/mL.

### Serum IL-33 measurement

IL-33 levels were determined using the sandwich ELISA kit. IL-33 concentrations were assayed according to the IL-33 ELISA protocol provided by the manufacturer (Human IL-33 ELISA Kit (Shanghai Sunred Biological Technology Co., Ltd, Baoshan District, Shanghai, China)). Briefly, 50 μL of standards and 40 fL of samples were added to the wells of 96-microwell plate and then 10 fL of IL-33 antibody was added to each of the sample wells. Additionally, 50 fL of Streptavidin-HRP was also added to each of the standard wells and each of the sample wells. The plate was then incubated for 1 hour at 37°C. Following incubation, each well was washed five times with 300 μL of wash buffer, and subsequently, 50 μL chromogen solution A and 50 μL chromogen solution B were added to each well and the plate was incubated in the dark at 37°C for 10 minutes. Then, 50 μL of stop solution was added to each well and the plate was measured with the absorbance at 450 nm with a Snergy HT plate reader (BioTek Instruments, Winooski, Vermont, USA). From the standard curve, the IL-33 levels in each test sample were quantitated.

### Serum sST2 measurement

IL-1RL1 levels were determined using the sandwich ELISA kit. IL-1RL1 concentrations were assayed according to the IL-1RL1 ELISA protocol provided by the manufacturer (BOSTER Immunoleader Human IL-1RL1 ELISA Kit (BOSTER Biological Technology Co., Ltd, Pleasanton, CA, USA)). Briefly, 100 μL of standards, samples (i.e., undiluted serum samples), and sample diluent (for zero, control, well) were added to the wells of a 96-microwell plate and incubated for 90 minutes at 37°C. After incubation, the liquid in each well was aspirated, and 100 μL biotinylated antibody was added to each well. The plate was then incubated for 60 minutes at 37°C. After this round of incubation, each well was washed three times with 300 μL of 0.01M PBS wash buffer. Then, 100 μL of the prepared ABC working solution was added to each well, and the plate was further incubated at 37°C for 30 minutes. After incubation, each well was washed five times with 300 μL of 0.01M PBS wash buffer and 90 μL of the prepared TMB colour developing agent was added to each well; the plate was then subsequently incubated in the dark at 37°C for 20 minutes. Then, 100 μL of prepared TMB stop solution was added to each well and the plate was measured with regards to the absorbance at 450 nm with a Snergy HT plate reader (BioTek Instruments, Winooski, Vermont, USA). From the standard curve, the IL-1RL1 levels in each test sample were quantitated.

### Assessment of endothelial function

Endothelium-dependent vasodilatation (i.e., flow-mediated dilatation (FMD)) and endothelium-independent vasodilatation (i.e., nitroglycerine-mediated dilatation (NMD)) of the brachial artery were assessed non-invasively, using high-resolution ultrasound as described by Celermajeret et al. [26]. Measurements were made by a single observer using an ATL 5000 ultrasound system (Advanced Technology Laboratories Inc., Bothell, WA., USA) with a 12-Mhz probe. The vascular assessment method was in agreement with the criteria set forth by the International Brachial Artery Reactivity Task Force [27]. All vasoactive medications were withheld for 24 hours before the procedure. The subjects also remained at rest in the supine position for at least 15 minutes before the examination started. Each subject’s right arm was comfortably immobilized in the extended position to allow for consistent recording of the brachial artery 2–4 cm above the antecubital fossa. Three adjacent measurements of end-diastolic brachial artery diameter were made from single 2D frames and all ultrasound images were recorded on Super Video Home System (S-VHS) videotape for subsequent blinded analysis. The FMD and NMD were then calculated as the percent change in diameter compared with baseline resting diameters. The intra-observer coefficient of variation for FMD was 5.9%.

### Statistical analyses

All statistical analyses were performed with the SPSS 19.0 (IBM, Armonk, NY, USA) statistical package. Non-normally distributed variables were expressed as median (range) and normally distributed variables were expressed as mean ± SD, where appropriate. A p value < 0.05 was considered to be statistically significant. Between-group comparisons were assessed for nominal variables with the Chi-square test and by the Kruskal-Wallis test (ANOVA) for the rest of variables. Spearman’s rank correlation was used to determine correlations between paired variables. Survival and time-to-event analysis of cardiovascular outcomes was performed using the Cox proportional hazards model, including the adjustment for potential confounding factors. Data is presented in the form of Hazard ratios (HR) and 95% confidence intervals (CI). Prevalence of fatal and nonfatal events according to IL-33 and sST2 categorical variables and estimated survival times for each category were calculated with Kaplan-Meier and Log Rank test.

## Results

This study included a total of 248 patients with different stages of CKD (i.e., 48 patients with stage 1, 53 patients with stage 2, 49 patients with stage 3, 49 patients with stage 4, and 49 patients with stage 5) and 49 healthy controls. The demographic data, laboratory findings, and endothelial function measurements of the patient and control groups are shown in Tables 1 and 2. The ages and genders of the patients were similar. Hypertension was higher in patients with stage 5 CKD, and the patients with stage 3–5 CKD had a history of cardiovascular disease. While systolic and diastolic blood pressures of the patient groups were similar, they were higher in the patient groups than those in the control group.

**Table 1:** Demographic and clinical characteristics of the study groups.

Parameters	Control	Stage 1(>90 ml/min) n:47	Stage 2 (60–89 ml/min) n:50	Stage 3 (30–59 ml/min) n:50	Stage 4 (15–29 ml/min) n:47	Stage 5 (0–14 ml/min) n:44	p
**Age (years)**	49(26–66)	48(26–69)	53(28–67)	49(27–69)	49(29–69)	49(26–69)	0.75
**eGFR(ml/min/1.73 m^2^)**	120(115–129)	95(91–107)	68(61–82)	44(30–89)	21(15–29)	5.5(7–14)	<0.001
**Presence of DM**		8	13	11	13	15	0.41
**Presence of HT**		5	10	11	6	22	<0.001
**Smoking (current)**		20	22	23	18	21	0.91
**History of CV disease(n)**							0.44
• Stroke		2	1	1	0	1	
• CVD		5	2	7	8	9	
• PVD		2	2	0	1	1	
• Aortic Aneurysm		1	0	0	0	0	
**Drugs**		26	25	20	20	16	0.36
• Calcium channel blocker		4	6	8	6	4	
• Beta blocker		1	2	0	0	0	
• Loop diuretic		0	0	1	0	1	
• ACE-i		8	2	10	6	10	
• ARB		5	11	7	11	9	
• Alpha blocker		0	1	2	1	2	
• Statin		3	3	2	3	2	
**SBP(mm Hg)**	130(119–139)	133(113–157)	134.5(120–163)	135(110–155)	132(113–175)	135(110–155)	0.03
**DBP(mm Hg)**	82(76–86)	82(71–93)	83(77–92)	85(80–95)	85(71–93)	83(70–95)	0.02

**Table 2:** Biochemical and vascular assessment according to CKD stages.

Parameters	Control	Stage 1(>90 ml/min) n:47	Stage 2 (60–89 ml/min) n:50	Stage 3 (30–59 ml/min) n:50	Stage 4 (15–29 ml/min) n:47	Stage 5 (0–14 ml/min) n:44	p
**Albumin(g/dl)**	4.2(3.5–5.10)	4(3.5–4.6)	3.8(3.5–4.6)	4.3(3.5–4.8)	4(3.4–4.6)	3(3.7–4.5)	<0.001
**Uric acid (mg/dl)**	4.1(3.2–5.8)	4.3(2.7–6.2)	4.7(2.7–6.8)	6.9(4.3–8.4)	7.5(4.2–9.7)	7.8(4–9.6)	<0.001
**Calcium (mg/dl)**	9.2(8.5–10)	8.9(8.3–10)	7.8(7.9–9.8)	8.3(7.5–9.2)	8(7–8.9)	8.1(7–8.6)	<0.001
**Phosphorus (mg/dl)**	3.9(2.7–4.4)	4.3(3.1–5.4)	4.3(3–6.7)	4.6(3.2–5.9)	5.4(4–8.9)	6.6(4.5–9.4)	<0.001
**PTH (pg/ml)**	47(21–65)	51(19–89)	59(21–121)	147(77–273)	142(52–218)	231(55–447)	<0.001
**Triglyceride. mg/dl**	137(115–167)	137(103–179)	139.5(106–159)	137(107–168)	138(124–202)	134(93–168)	0.04
**LDL Cholesterol (mg/dl)**	119(97–140)	126(85–161)	128(96–163)	121(96–152)	128(98–161)	122(81–171)	0.02
**HDL Cholesterol (mg/dl)**	44(27–53)	46(31–66)	44(26–54)	42(26–50)	43(28–63)	43(26–59)	0.10
**Total Cholesterol (mg/dl)**	192(159–265)	194(160–254)	193(170–235)	193.5(171–235)	194(159–253)	192(149–235)	0.64
**Proteinuria (g/day)**	0.08 (0.04–0.15)	1.3 (0.2–3.8)	1.4 (0.2–5.0)	1.5 (0.4–5.3)	1.6 (0.3–5.3)	1.2 (0.2–2.5)	<0.001
**Hemoglobin (g/l)**	15(12–17)	13.4(7.4–17)	12.0(8–16.9)	12.3(7.4–16)	11.5(8.8–16.8)	10.7(7–16.6)	<0.001
**Hs-CRP (mg/l)**	2(1–4)	7.6(3.2–16)	10(5–24)	16(5–35)	23(4.7–46)	27.5 (4–64)	<0.001
**IL33 (ng/l)**	39(26–168)	72.8(28–168)	109.7(24.7–199)	281.2(57.2–528)	400(31.3–1226)	526.19(44.7–1230)	<0.001
**sT2 (pg/ml)**	245(115–469)	341(170–580)	355.2(187–590)	389.5(247–780)	540(324.4–1130)	718.3(220–1320)	<0.001
**Homa-IR index**	1.34(.90–2.06)	1.380(95–3.28)	1.465(1.01–2.92)	1.52(.97–7.45)	1.72(1.9–1.62)	1.71(1.14–5.68)	0.01
**FMD. %**	9(7.5–11.2)	8.2(7.2–9.7)	7.2(6.2–8.3)	6.8(5.8–8.2)	6.2(4.1–8.2)	5.35(4–7.2)	<0.001
**CV disease**		5	13	13	17	20	0.004
**Exitus**							0.17
**• CVD associated**		0	2	3	2	5	
**• Other**		0	2	1	0	0	

Upon examining the laboratory values, with respect to the increasing stage of CKD, it was observed that albumin and haemoglobin levels decreased, but uric acid, phosphorus, PTH levels, Hs-CRP, IL-33, and ST2 levels increased. Moreover, HOMA-IR index was increased with increasing stage of CKD **(Table 2)**.

In a correlation analysis, IL-33 and ST2 levels were found to be correlated with FMD, eGFR, haemoglobin, Hs-CRP, HOMA-IR, uric acid, Ca, P, and PTH (Table 3).

**Table 3:** Univariate associates of IL 33 and ST2 in non-dialysis CKD patients.

	IL 33		ST2	
	rho	p value	rho	p value
**GFR**	-0.62	<0.001	-0.56	<0.001
**proteinuria**	0.11	0.07	0.03	0.58
**HOMA**	0.24	<0.001	0.12	0.06
**Hs-CRP**	0.64	<0.001	0.43	<0.001
**Uric acid**	0.50	<0.001	0.40	<0.001
**Age**	-0.10	0.11	0.06	0.39
**Ca**	-0.40	<0.001	-0.40	<0.001
**P**	0.37	<0.001	0.38	<0.001
**PTH**	0.58	<0.001	0.51	<0.001
**Hgb**	-0.23	<0.001	-0.14	0.03
**FMD**	-0.46	<0.001	-0.48	<0.001
**IL 33**	-	-	0.41	<0.001
**ST2**	0.41	<0.001	-	-

### Endothelial functions

Endothelial functions were also found to deteriorate with the increasing stage of CKD. In the univariate correlation, FMD showed a positive correlation with eGFR (p < 0.001) and a negative correlation with systolic blood pressure (p = 0.01), HOMA (p < 0.001), PTH (p < 0.001), Hs-CRP (p < 0.001), IL-33 (p < 0.001), and ST2 (p < 0.001). In linear regression analysis, systolic blood pressure, CRP, ST2, and PTH were found to be a predictor for FMD.

### Follow-up period

During the 24-month follow-up period, while nonfatal cardiovascular events were seen in 56 patients (i.e., coronary heart disease (n = 32), sudden death (n = 4), stroke (n = 17), and/or complicated peripheral vascular disease (n = 4)), fatal cardiovascular events were seen in 12 patients (i.e., coronary heart disease (n = 6), sudden death (n = 2), stroke (n = 3), or complicated peripheral vascular disease (n = 1)). The predictors for time-to-CV event (fatal and nonfatal CV events = 68) were studied via univariate and multivariate Cox regression analyses. We included all significant parameters derived from the univariate analysis and well-known risk factors for CV disease (such as age and sex) into the multivariate Cox model. The multivariate Cox analysis showed that the presence of diabetes mellitus, smoking, and proteinuria and levels of haemoglobin, Hs-CRP, IL-33, and ST2 were associated with the risk of CV events (Table 4).

**Table 4:** Univariate and multivariate COX analysis predicting for cardiovascular outcomes (a composite of 68 fatal and non-fatal CV events).

	Unvariable (Unadjusted HR)		Multivariable (Adjusted HR)	
**IL 33 (ng/l)**	1.003 (1.002–1.004)	<0.001	1.002(1.001–1.003)	0.003
**sT2 (pg/ml)**	1.003 (1.002–1.003)	<0.001	1.002 (1.00–1.003)	0.007
**hsCRP (mg/l)**	1.07 (1.04–1.09)	<0.001	1.03 (1.00–1.067)	0.01
**Diabetes (yes/no)**	4.20 (2.61–6.77)	<0.001	2.14(1.27–3.60)	0.004
**Smoking (yes/no)**	1.84 (1.14–2.97)	0.01	1.71(1.04–2.86)	0.03
**Hb (g/l)**	1.15 (1.03–1.29)	0.01	1.27(1.11–1.46)	<0.001
**Proteinuria**	1.00 (1.00–1.01)	0.001	1.02(1.01–1.03)	0.005

Represented are Hazard Ratios (and 95% confidence intervals) in univariate (crude) Cox model and after different adjustments. Variables included in to model: age, gender, diabetes history, smoking, systolic blood pressure, diastolic blood pressure, glomerular filtration rate, flow mediated dilatation, The Homeostasis Model Assessment, albumin, hemoglobin, calcium, phosphorus, parathyroid hormone, lipid parameters, hs-CRP, IL 33 and ST2 (Model chi-square:15)

**Fig 1:**
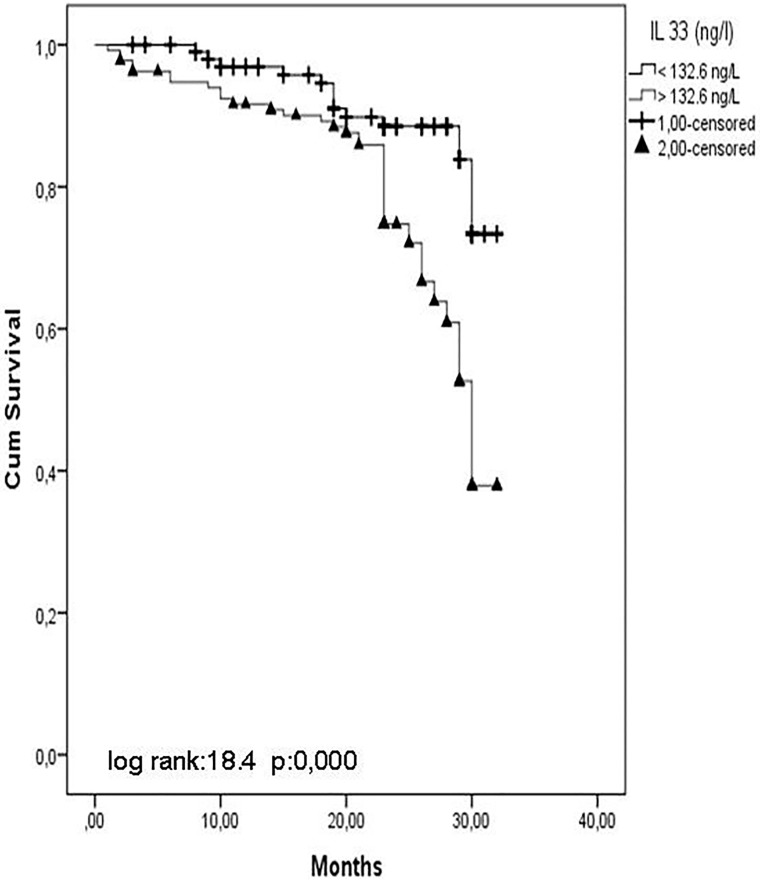
Kaplan–Meier survival curves showed that patients with IL 33.

**Fig 2:**
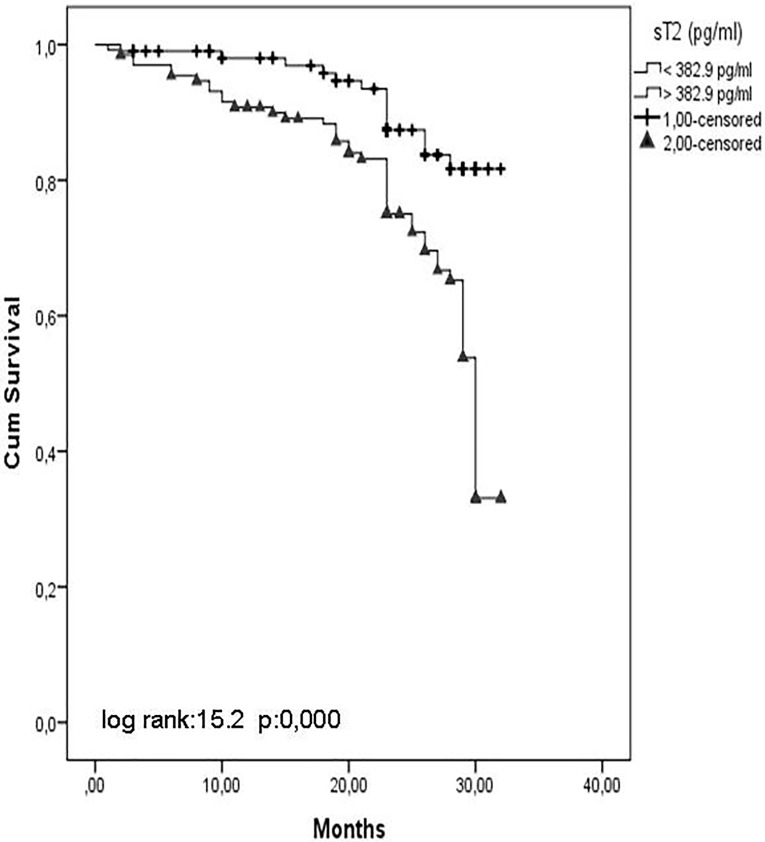
Kaplan-Meier survival curves showed that patients with ST2.

In addition, Kaplan-Meier survival curves showed that patients with IL-33 and ST2 values below the median value (IL-33 = 132.6 ng/L, ST2 = 382.9 pg/ml) had a higher cumulative survival rate as compared with patients who had IL-33 and ST2 levels above the median value (log-rank test, p < 0.001) (Figs 1 and 2).

### Prediction of cardiovascular outcomes

Cardiovascular outcomes were determined from the time of patient inclusion in the study, with a mean follow-up period of 24 (range 1 to 32) months. Fifteen patients died, 12 of them were due to presumable cardiovascular causes. Cardiovascular mortality was defined as death due to coronary heart disease (n = 6), sudden death (n = 2), stroke (n = 3), or complicated peripheral vascular disease (n = 1).

A total of 56 additional nonfatal major adverse cardiovascular events took place during the follow-up period. These included stroke (n = 28), myocardial infarction (n = 26), and peripheral vascular disease (n = 2). The predictors for time-to-cardiovascular event (fatal and nonfatal cardiovascular events = 68 (56 nonfatal + 12 fatal CVE)) were studied by univariate and multivariate cox regression analyses.

## Discussion

In this prospective study, we found that IL-33 and ST2 levels increased with increasing stage of CKD in patients with chronic renal failure and IL-33, ST2, hs-CRP, diabetes and smoking history, proteinuria and Hb level were associated with vascular dysfunction and were also a predictor of fatal–nonfatal cardiovascular events and survival.

Inflammation must be accepted as the protective attempt by the organism to remove the injurious stimuli [28]. It also has the purpose of starting the healing process in the tissues. While proinflammatory cytokine release might have useful influences in the organism, it is also possible that there are detrimental effects due to chronic systemic inflammation. In fact, this is the problematic situation observed in CKD. As it is well-known, CKD is a multifactorial disorder. It occurs in the context of chronic co-morbidities like glomerulonephritis, hypertensive nephropathy, and diabetes nephropathy. Several studies have been conducted, and it has been reported that CKD is known to be an immune-inflammatory condition [29,30]. A persistent low-grade inflammation is often observed in these patients [3]. Systemic concentrations of pro-/anti-inflammatory cytokines are several times higher in individuals with CKD, which is the result of decreased renal clearance and increased production [31]. Certain cytokines (such as IL-12 and IL-18), which are implicated in immune inflammation, are also known to be active in the development of CKD [32,33]. This situation leads to the disruption of the cells and tissues and it has deleterious effects on the body.

The IL-1 family has several inflammatory cytokines, including IL-1α, IL-1β, and IL-18. It also plays an important role in inflammatory diseases as well as in infectious and autoimmune diseases [34]. In the IL-1 family, interleukin-33 (IL-33) is a new cytokine. Some normal tissues may express IL‑33 in a constitutive and widespread manner. It also mediates some biological influences by interacting with receptors, the suppression of tumorigenicity 2 (ST2) and IL‑1 receptor accessory protein [35]. IL-33 behaves like a dual function molecule (i.e., a nuclear factor and a cytokine) and promotes the activation of nuclear factor (NF)-κB and mitogen-activated protein kinase by binding to the ST2 receptor. This leads to the increased transcription of Th2 cytokines [36]. It appears that the IL-33/ST2 axis has a crucial role in some chronic immune inflammatory disorders, such as asthma, rheumatoid arthritis, and anaphylactic shock [36–38]. In recent years, the levels and functions of IL-33 and ST2 in renal diseases have been the subject of research. In a study performed in mice by Akcay et al., it was shown that IL-33 increased acute renal damage due to cisplatin [39]. In a study conducted in patients with CKD, Bao et al., compared patients with CKD with healthy controls and found that while IL-33 levels were similar in both groups, ST2 levels were higher in patients with CKD than that in the healthy controls [40]. In our study, IL-33 and ST2 levels were higher in patients with CKD than that in healthy controls, and IL-33 and ST2 levels increased progressively with increasing stages of CKD. From this perspective, our study is the first of its kind in literature. In our study, as in previous studies, Hs-CRP level was increased with the increasing stage of CKD. Moreover, an increase in these three inflammatory markers, along with the increasing stage of CKD, supports progressive inflammation in CKD.

### IL-33, ST2, and endothelial dysfunction

The most important cause of death was cardiovascular events in CKD and end-stage renal failure. Endothelial dysfunction occupies an important place in the pathogenesis of cardiovascular events. Diminished nitric oxide (NO) production or availability and/or imbalance of relative contribution of endothelium-derived relaxing-contracting factors, like endothelin-1 (ET-1), angiotensin, and oxidants, are associated with endothelial dysfunction. The reason(s) for endothelial dysfunction in patients with CKD are not fully understood, but are probably multifactorial [41]. Endothelial dysfunction is closely related to inflammation. It is known that cell‐surface adhesion molecules are expressed after the endothelial cells are exposed to pro‐inflammatory cytokines [42]. This also impairs the endothelium‐dependent vascular relaxation [42]. Fichtlscherer et al. conducted a study and showed that there is an inverse correlation between the levels of CRP and the forearm blood flow of acetylcholine in male patients with coronary artery diseases. It is important that the study found that the CRP levels decreased in time and that this is associated with the normalization of endothelium-mediated forearm blood flow responses in three months [43]. Cleland et al. conducted a study and reported that there is a relation between the low-level inflammation and basal endothelial NO synthesis [44]. In recent years, IL-33 has been the focus of interest to have a relationship with endothelial functions. In a study performed by Chalubinski et al., they showed that IL-4 and IL-33 destroyed human microvascular endothelium in different ways [45]. IL-33 and IL-4 caused the endothelial integrity to decrease and the permeability to increase. When IL-33 and IL-4 were added together, they caused the endothelial integrity to decrease by two-fold than when they were administered separately [45]. Down-regulation of occluding and VE-cadherin mRNA expression accompanies this effect. In addition, IL-4 induced cell apoptosis, but IL-33 did not. IL-33 and IL-4 showed the additive potency to down-regulate VE-cadherin mRNA expression [45]. Unlike IL-4, IL-33 increased the ICAM-1 surface expression (but not PECAM-1) in endothelial cells [45]. As such, flow-mediated dilatation measurement is a reliable indicator of endothelial dysfunction. In our study, endothelial functions deteriorated with increasing stage of CKD, and this is consistent with reports in literature. Additionally, in our study, FMD showed a positive correlation with eGFR and a negative correlation with systolic blood pressure, HOMA, PTH, Hs-CRP, IL-33, and ST2. In linear regression analysis, systolic blood pressure, CRP, ST2, and PTH were found to be predictors. In our study, it was also shown for the first time that IL-33 and ST2 had an effect on endothelial dysfunction in patients with kidney disease; these findings also support other patient populations.

### IL-33 and cardiovascular events and death

Inflammation and proinflammatory cytokines play an important role in the development and progression of cardiovascular events [46]. These factors can induce myocardial remodelling by causing the recruitment of inflammatory cells and/or by showing maladaptive effects such as endothelial dysfunction on left ventricular remodelling, and thus, may promote hypertrophy and fibrosis [47]. In recent years, it has been reported that there is IL-33 expression in coronary artery smooth muscle cells, in coronary artery endothelium, endothelial cells, and in cardiac fibroblasts, and that ST2 is expressed by endothelial cells in cardiac vessels [48]. TNF-α, IFN-γ, and IL-1β are known to upregulate IL-33. Additionally, IL-33 is expressed during the necrosis of human cardiac and smooth muscle cells. Studies have been conducted in recent years and have reported that IL-33/ST2 has an active role in cardiovascular diseases [49,50]. It is also influential in the protection of cardiac muscle. In recent years, it has been the subject of much research that IL-33 and ST2 are associated with acute myocardial infarction, acute and chronic heart failure, aortic stenosis, and diastolic dysfunction, and interestingly, increased ST2 levels were generally found to be associated with poor outcomes [51–53]. Moreover, it has been suggested that ST2 levels can be used as a cardiac biomarker [54]. There were few studies conducted regarding IL-33/ST2 and cardiovascular risk in patients with kidney disease. In a study conducted in 95 renal transplant patients by Mansell et al., they found that IL-33 levels were increased in patients with high cardiovascular risk as compared with patients with low cardiovascular risks [55]. In our study, it was found that increased IL-33 and ST2 levels were associated with endothelial dysfunction, ST2 was one of the predictors of endothelial dysfunction, and both IL-33 and ST2 were predictors of fatal and nonfatal cardiovascular events during the follow-up period of the patients. This study is the first study to show that the IL-33/ST2 pathway is associated with increased cardiovascular events and poor outcomes in non-dialysis chronic kidney disease patients. The inflammatory process is enhanced with deterioration in renal functions in patients with chronic kidney disease. Therefore, the association of increased inflammation with endothelial dysfunction and poor cardiovascular outcomes in these patients may be mediated by IL-33/ST2 activation. The inhibition of this activation or its blockade at the receptor level may help prevent cardiovascular events.

Consequently, traditional risk factors and increased IL-33/ST2 levels in patients with chronic kidney disease are an indicator of increased inflammation, impaired endothelial functions, and cardiovascular events.
